# Effects of an Isolate of *Bacillus subtilis* on Southern Blight Severity of *Aconitum carmichaelii* Debeaux and Its Rhizosphere Microbial Community

**DOI:** 10.3390/plants15152308

**Published:** 2026-07-27

**Authors:** Xiaofang Sun, Yong Liu, Pengsheng Ye, Lian He, Shundong Dai, Zaiyin Kuang, Qiuping Jiang, Hualan Zeng

**Affiliations:** Industrial Crop Research Institute, Sichuan Academy of Agricultural Sciences, Chengdu 610300, China

**Keywords:** rhizosphere microbiome, *Aconitum carmichaeli*, southern blight, *Bacillus subtilis*, biological control

## Abstract

Southern blight caused by *Sclerotium rolfsii* is a devastating soil-borne disease of *Aconitum carmichaelii*, a medicinal plant widely cultivated in China. Biological control using beneficial bacteria offers a sustainable alternative to chemical fungicides. This study investigated the influence of *Bacillus subtilis* FZ-7 on rhizosphere microbiome assembly and disease suppression in *A. carmichaelii* under field conditions. Four treatments were established: uninoculated control (C group), single inoculation with *B. subtilis* (B group), single inoculation with *S. rolfsii* (S group), and co-inoculation of *S. rolfsii* and *B. subtilis* (BS group). Plant biomass, soil physicochemical properties, and rhizosphere bacterial and fungal communities were analyzed using high-throughput sequencing of 16S rRNA and ITS genes, along with co-occurrence network analysis. Results showed that *B. subtilis* inoculation suppressed this growth suppression. *B. subtilis* alone enhanced both bacterial and fungal diversity. PCoA and LEfSe analyses revealed distinct microbial community structures among treatments, with biomarkers such as *Lactobacillus*, *Candidatus Nitrosotalea*, *Rhodanobacter*, and *Sphingomonas* for bacteria, and *Trichocladium*, *Conocybe*, *Plectosphaerella*, and *Athelia* for fungi. Co-occurrence network analysis indicated that co-inoculation of *S. rolfsii* and *B. subtilis* partially restored cooperative microbial associations. Soil organic matter, pH, and cation exchange capacity were the main environmental factors shaping microbial community composition. Shoot fresh weight in BS recovered to 2.98 kg and rhizome fresh weight to 3.72 kg, representing 73.0% and 41.2% increases over the S treatment. Additionally, the disease index was reduced by 55.26 in BS compared with the control group. Nay more, *Bacillus*, *Chujaibacter*, and *Rhodanobacter* taxa were significantly enriched in BS group. In conclusion, *B. subtilis* FZ-7 effectively mitigates southern blight in *A. carmichaelii* by modulating rhizosphere microbial assembly, enriching beneficial taxa, and stabilizing microbial co-occurrence networks, highlighting its potential as a biocontrol agent for sustainable medicinal plant production.

## 1. Introduction

*Aconitum carmichaelii* Debeaux (family Ranunculaceae) is a perennial medicinal herb highly valued in traditional Chinese medicine. The processed lateral roots, known as ‘Fuzi’, are rich in diterpene alkaloids and are widely used to treat cardiovascular diseases, rheumatoid arthritis, and various inflammatory disorders [1,2]. The cultivation of *A. carmichaelii* has a history of over a thousand years in China, particularly in Sichuan, Yunnan, and Shaanxi provinces, where it serves as a major cash crop for local farmers [3]. However, the yield and quality of *A. carmichaelii* are severely threatened by soil-borne diseases, especially southern blight and root rot, which can cause annual yield losses of 60–80% in Sichuan province [4].

Southern blight, caused by the necrotrophic fungus *Sclerotium rolfsii* (teleomorph: *Athelia rolfsii*), is characterized by wilt, root rot, and the presence of white mycelia and brown sclerotia at the stem base or on roots [5]. *S. rolfsii* produces persistent sclerotia that can survive in soil for years, making disease management particularly challenging [6]. Root rot, on the other hand, is primarily attributed to *Fusarium oxysporum*, which colonizes the vascular system and leads to systemic wilting and plant death [4,7]. Currently, farmers heavily rely on chemical fungicides such as carbendazim and fludioxonil to control these diseases. However, the overuse of chemical pesticides raises serious concerns regarding environmental pollution, food safety, and the development of resistant pathogen populations [8].

In response to these challenges, biological control using beneficial microorganisms has emerged as a promising and sustainable alternative [9]. Plant endophytic bacteria, in particular, have gained attention because they live inside host tissues, are well-adapted to the plant environment, and can suppress pathogens through multiple mechanisms, including the production of antifungal metabolites, lytic enzymes, volatile organic compounds, and induction of systemic resistance in the host [5,10]. Among them, species of the genus Bacillus are among the most studied biocontrol agents due to their broad-spectrum antagonistic activity, ability to form heat-resistant spores, and plant growth-promoting properties [8,11].

Recent studies have demonstrated that *Bacillus velezensis* and *Bacillus subtilis* strains can effectively suppress soil-borne fungal diseases in various crops. For instance, *B. velezensis* YN-2-6S, isolated from the stem of healthy *A. carmichaelii*, exhibited strong inhibitory effects against *S. rolfsii* and *F. oxysporum* both in vitro and in field trials, achieving biocontrol efficiencies of 91.00% and 85.71% against southern blight and root rot, respectively [12]. Similarly, *B. subtilis* has been reported to promote plant growth and induce systemic resistance in many plant species. However, the specific influence of *B. subtilis* inoculation on the assembly and function of the rhizosphere microbiome of *A. carmichaelii*, particularly under *S. rolfsii* pressure, remains poorly understood.

The rhizosphere microbiome plays a critical role in plant health by shaping nutrient cycling, suppressing pathogens, and modulating plant defense responses [13]. Pathogen infection can dramatically alter the composition and co-occurrence patterns of rhizosphere microbial communities, often leading to the enrichment of beneficial taxa that help the plant combat the disease [14]. Conversely, continuous monocropping and disease outbreaks can destabilize microbial networks and reduce the soil’s natural disease-suppressive capacity [15]. Therefore, understanding how biocontrol agents like *B. subtilis* reshape the rhizosphere microbiome and restore microbial balance is essential for developing more effective and ecologically sound disease management strategies.

In the initial phase, a total of 85 isolates were obtained from healthy *A. carmichaelii* rhizosphere. The strain FZ-7 showed the strongest inhibition in dual-culture assays (inhibition zone 18.3 ± 1.2 mm, relative inhibition 76.4%), demonstrating significant inhibitory activity against *S. rolfsii* in vitro. Accordingly, the present study was designed to: (1) evaluate the effect of *B. subtilis* FZ-7 on the growth and severity of southern blight disease in *A. carmichaelii* under field conditions; (2) investigate the changes in rhizosphere soil physicochemical properties and microbial community composition (bacterial and fungal) following *S. rolfsii* infection and *B. subtilis* inoculation using high-throughput sequencing; (3) analyze the co-occurrence networks of rhizosphere microorganisms to understand how *B. subtilis* modulates microbial interactions and community stability; and (4) identify potential microbial biomarkers associated with disease suppression. The results will provide a theoretical basis for the ecological control of southern blight in *A. carmichaelii* and facilitate the development of *Bacillus*-based biocontrol products for sustainable medicinal plant production.

## 2. Results

### 2.1. Effect of Southern Blight on the Yield and Disease Index of A. carmichaeli

**Figure 1:**
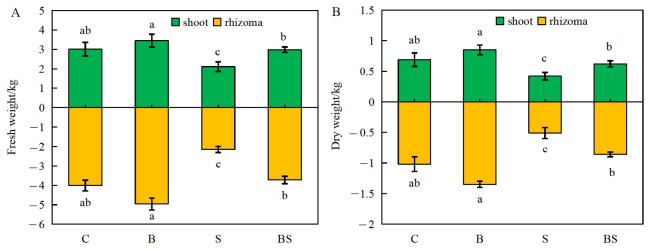
Effects of southern blight on the fresh weight (**A**) and dry weight (**B**) of both the shoot and rhizoma in *A. carmichaeli*. Different lowercase letters positioned above the error bars indicate statistically significant differences (*p* < 0.05; Duncan’s test).

Compared with the uninoculated control (C), single inoculation with *B. subtilis* (B) significantly promoted the growth of *A. carmichaelii*, increasing shoot fresh weight from 3.01 kg to 3.45 kg, rhizoma fresh weight from 4.01 kg to 4.96 kg, shoot dry weight from 0.69 kg to 0.85 kg, and rhizoma dry weight from 1.02 kg to 1.35 kg, respectively (Figure 1). In contrast, inoculation with *S. rolfsii* alone (S) severely inhibited plant growth, resulting in substantial reductions in both fresh and dry biomass: shoot fresh weight decreased to 2.11 kg, rhizoma fresh weight to 2.15 kg, shoot dry weight to 0.42 kg, and rhizoma dry weight to 0.51 kg, representing declines of 29.9%, 46.4%, 39.1%, and 50.0% relative to the control, respectively. Notably, co-inoculation with *B. subtilis* and *S. rolfsii* (BS) effectively mitigated the pathogen-induced growth suppression. Compared with the *S. rolfsii*-only treatment, BS increased shoot fresh weight by 41.2% (to 2.98 kg) and rhizoma fresh weight by 73.0% (to 3.72 kg), while shoot and rhizoma dry weights recovered to 0.62 kg and 0.86 kg, respectively, levels that were close to those observed in the uninoculated control.

No disease symptoms were observed in the blank control group (C) and the single *B. subtilis* inoculation group (B), with both disease incidence and disease index maintained at 0 (Table 1, Figure S1). In the group (S) only inoculated with *S. rolfsii*, the disease incidence reached 100%, accompanied by a high disease index of 76.29. For the co-inoculation group (BS) treated with both strains, disease incidence was reduced to 36.22% and disease index dropped to 21.03, achieving a relative disease control efficiency of 72.43%. Significant differences were detected among groups, which verified that *B*. *subtilis* could effectively suppress the disease damage induced by *S. rolfsii* and exert prominent biocontrol potential against aconite southern blight.

### 2.2. General Characteristics of Illumina Novaseq Pe250 Sequencing Results

**Figure 2:**
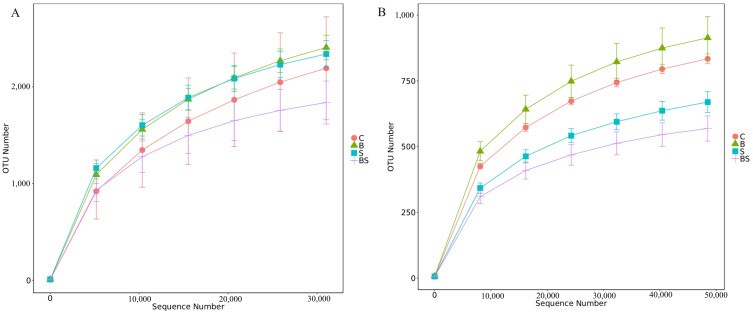
Rarefaction curves for rhizosphere soil bacteria (**A**) and fungi (**B**).

A total of 24 soil samples were distinguished and analyzed based on barcode-labeled amplicon sequences. For fungal identification, 2,067,807 circular consensus sequencing (CCS) reads were generated across all samples, with individual samples yielding 78,274 to 93,074 reads (mean = 86,159). For bacterial taxonomic annotation, 1,510,624 CCS reads were recovered, ranging from 52,455 to 82,629 reads per sample (mean = 62,942). After quality filtering, barcode processing, and chimera removal, the proportion of high-quality effective reads exceeded 98%. On average, each sample produced 65,962 optimized fungal CCS reads, of which more than 95% exhibited a quality score ≥ 30. For bacteria, the average number of optimized CCS reads per sample was 46,866, with an effective rate of 97% and over 91% of sequences scoring ≥ Q30. These metrics confirmed that the sequencing data were sufficiently reliable and robust for downstream bioinformatic analysis. Rarefaction curves for both bacterial and fungal OTUs plateaued at approximately 20,000 sequences per sample, indicating that the sequencing depth adequately captured the majority of rhizosphere microbial diversity (Figure 2).

### 2.3. Venn Diagrams Analysis of Otus

**Figure 3:**
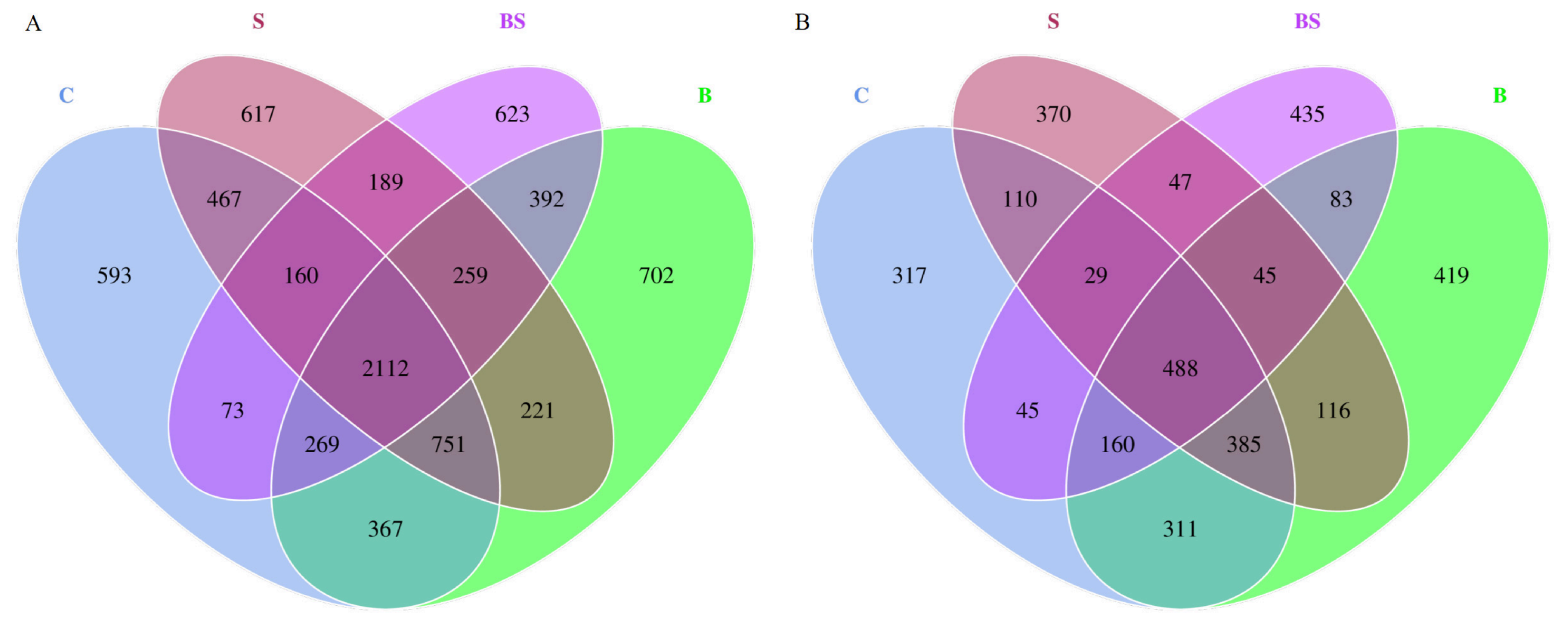
OTU Venn diagram analysis of rhizosphere soil bacteria (**A**) and fungi (**B**).

On average, the 24 soil samples yielded 7795 bacterial operational taxonomic units (OTUs) and 3360 fungal OTUs, with coverage rates exceeding 97% and 98%, respectively. These values demonstrated that the sequencing depth was sufficient to support reliable downstream analyses of the rhizosphere microbial communities. Venn diagrams were constructed to intuitively display OTU distributions across healthy and diseased samples, highlighting both common and unique taxa. For bacteria, 2112 OTUs were shared across all four treatments (Figure 3A), representing the core rhizosphere microbiome. The greatest number of unique bacterial OTUs was detected in the B treatment (702), followed by BS (623), S (617), and C (593). Regarding fungal communities (Figure 3B), 488 OTUs were shared by all four groups. The highest count of unique fungal OTUs appeared in the B treatment (419), followed by BS (435), S (370), and C (317). Collectively, these findings demonstrated that each treatment maintained a distinct microbial assemblage composed of both exclusive and shared taxa. Notably, the BS co-inoculation group possessed abundant unique OTUs, implying that the combined interaction between *B. subtilis* and *S. rolfsii* reshaped the rhizosphere microbial structure. Meanwhile, the large number of shared OTUs suggests a relatively stable core community in the rhizosphere of *A. carmichaelii*, treatment-specific unique OTUs show clear shifts beyond this core.

### 2.4. Alpha-Diversity Analysis of Rhizosphere Soil Microbial Community

**Figure 4:**
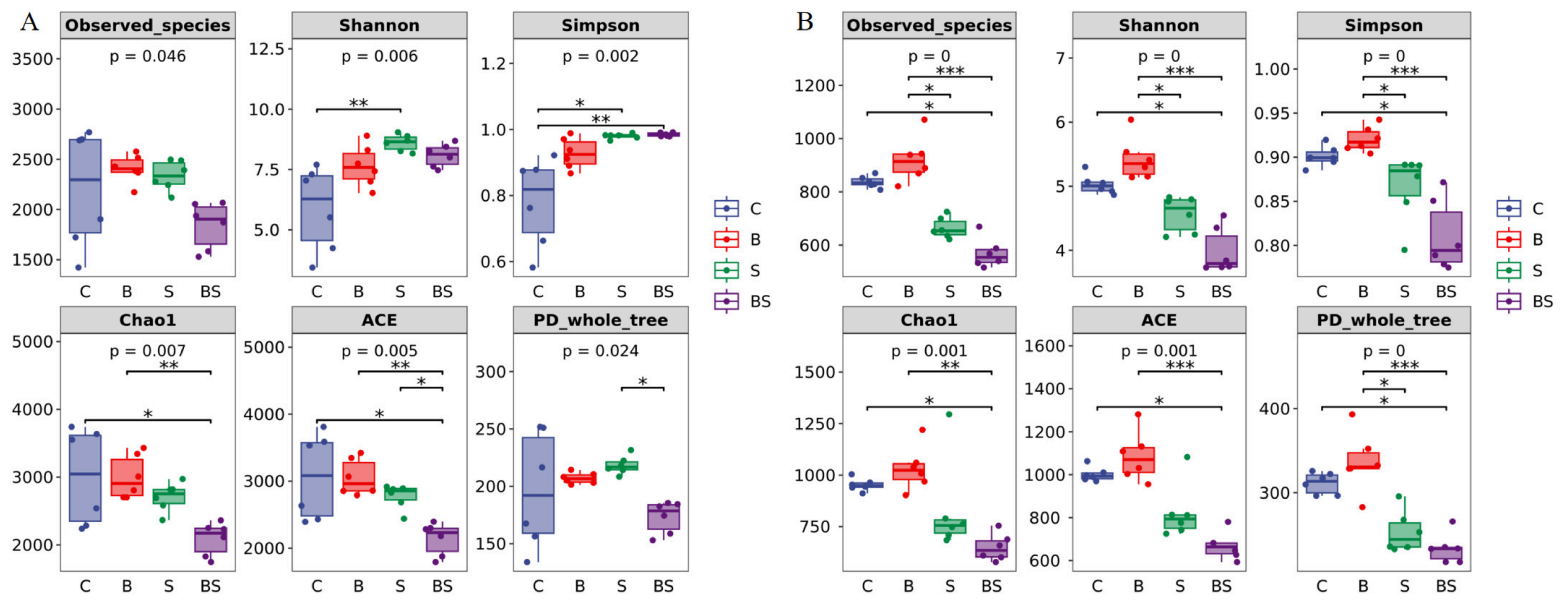
Alpha-diversity indexes between the healthy and diseased plants rhizosphere soil bateria (**A**) and fungi (**B**). * *p* < 0.05, ** *p* < 0.01, *** *p* < 0.001.

Alpha-diversity analysis was conducted to evaluate the abundance, variety, and uniformity of bacteria and fungi present in the rhizosphere soil. For bacterial communities (Figure 4A), the alpha diversity indices showed significant differences among treatments: the Simpson, Shannon, Chao1, and ACE indices showed highly significant differences (*p* < 0.01), and the observed species and PD_whole_tree indices showed significant differences (*p* < 0.05). The single *S. rolfsii* inoculation group had the highest bacterial evenness, with the Shannon and Simpson indices reaching 8.61 and 0.98, respectively, which were significantly higher than those in the control group. The single *B. subtilis* inoculation group also showed significantly higher bacterial diversity than group C, while the co-inoculation group BS had the lowest bacterial richness and phylogenetic diversity, with the observed species and PD_whole_tree decreasing to 1840.83 and 173.13, respectively. For fungal communities (Figure 4B), all six alpha diversity indices also showed extremely significant differences among the four treatments (*p* < 0.001). Unlike the trend in fungal communities, compared with the control group, the single *B. subtilis* inoculation group exhibited significantly higher fungal richness, evenness, and phylogenetic diversity, with the observed species increasing from 837.33 to 922.00, the Shannon index rising from 5.03 to 5.43, and the PD_whole_tree increasing from 311.32 to 336.41. In contrast, the single *S. rolfsii* inoculation group showed a significant reduction in all fungal alpha diversity indices compared with group C, and the co-inoculation group had the lowest fungal diversity among all treatments, with the observed species, Shannon index, and PD_whole_tree decreasing to 569.00, 3.99, and 233.79, respectively.

Collectively, these results demonstrate that inoculation with *B. subtilis* and/or *S. rolfsii* significantly altered the alpha diversity of both rhizosphere fungal and bacterial communities in *A. carmichaelii*, with fungal communities being more sensitive to the co-inoculation treatment and bacterial communities showing a more pronounced response to single pathogen inoculation.

### 2.5. Rhizosphere Microbial Community Assembly

**Figure 5:**
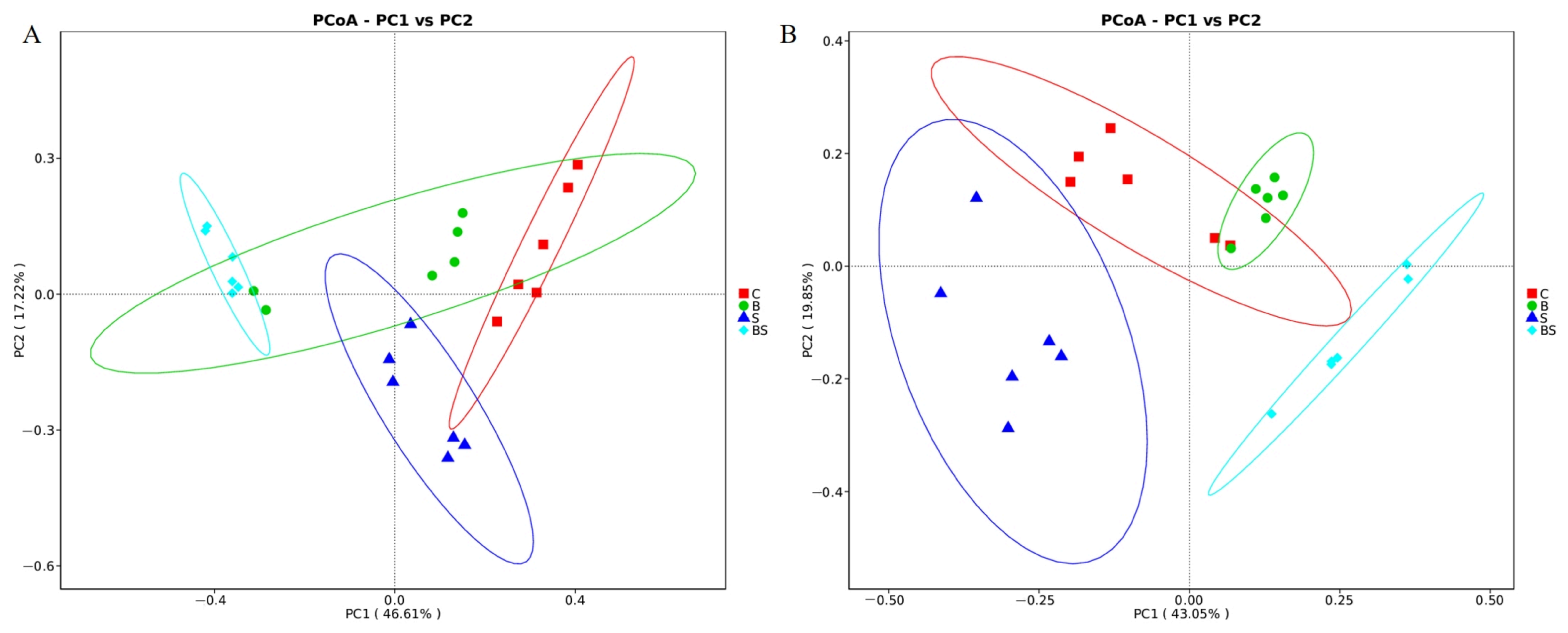
Principle co-ordinates analysis of dimension reduction analysis based on Bray-Curtis distances according to the abundance of rhizosphere bacteria (**A**) and fungi (**B**).

Principal Coordinate Analysis (PCoA) based on Bray-Curtis dissimilarity was performed to assess the effects of inoculation treatments on the overall structure of rhizosphere microbial communities in *A. carmichaelii*. For bacterial communities (Figure 5A), the first two principal coordinates (PC1 and PC2) explained 46.61% and 17.22% of the total variation, respectively, with clear separation of samples according to treatment. The uninoculated control, *B. subtilis*-inoculated, *S. rolfsii*-inoculated, and co-inoculated groups formed distinct clusters, indicating significant differences in bacterial community composition among treatments. Similarly, for fungal communities (Figure 5B), PC1 and PC2 accounted for 43.05% and 19.85% of the variation, respectively. Samples from each treatment were clearly separated in the ordination space, with the C, B, S, and BS groups forming distinct, non-overlapping clusters. These results demonstrate that inoculation with *B. subtilis* and/or *S. rolfsii* significantly altered the overall structure of both rhizosphere bacterial and fungal communities, with each treatment driving the formation of a distinct microbial assemblage.

**Figure 6:**
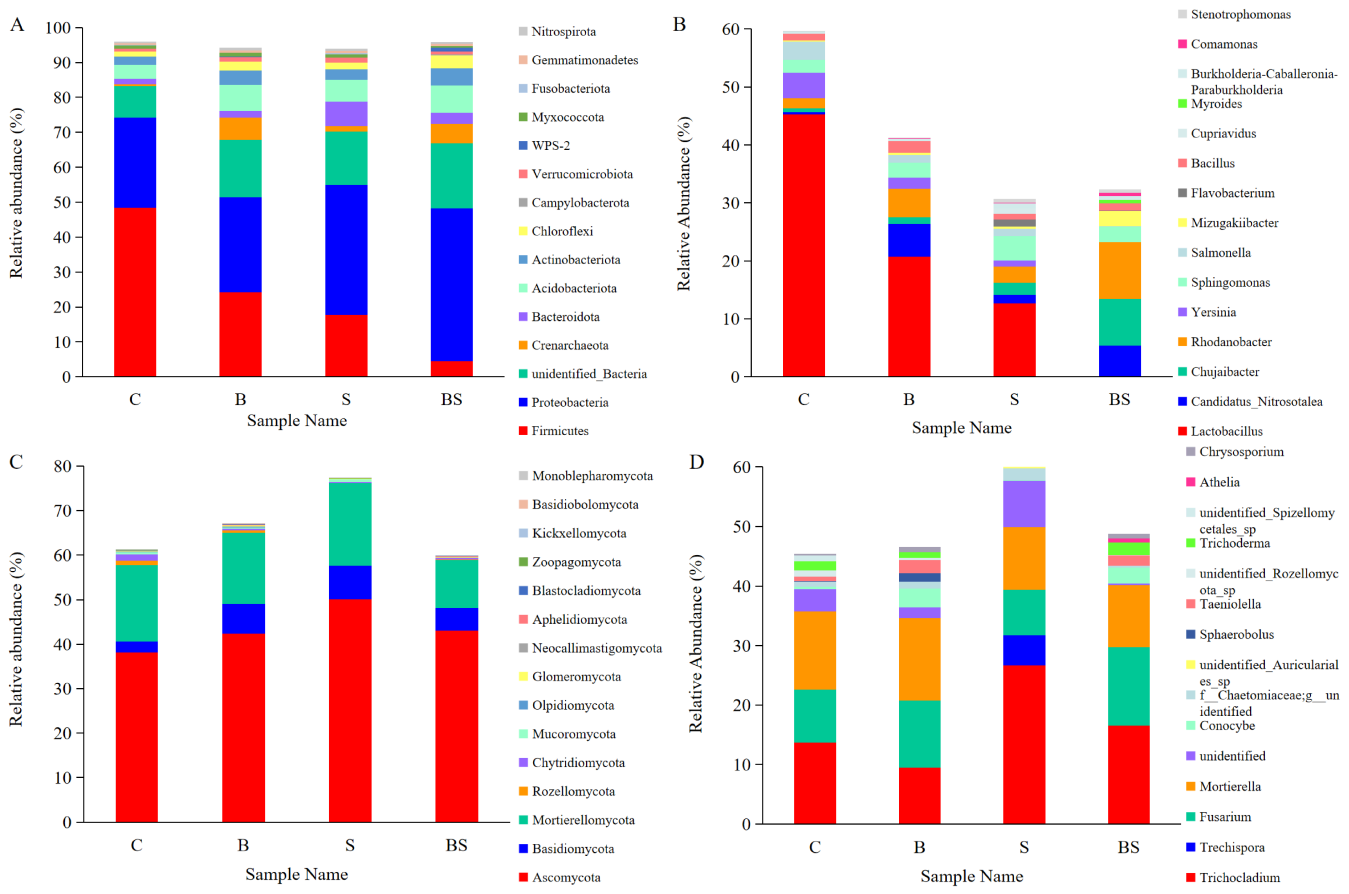
Taxonomic summary of the relative abundance of the rhizosphere bacterial phyla (**A**), genera (**B**) and fungal phyla (**C**), genera (**D**) in healthy and diseased soil.

The taxonomic composition of rhizosphere bacterial and fungal communities was analyzed at the phylum and genus levels across the four treatments. At the bacterial phylum level (Figure 6A), Firmicutes and Proteobacteria were the dominant phyla across all treatments, together accounting for over 70% of the total relative abundance. Compared with the control, the relative abundance of Firmicutes decreased significantly in treatments inoculated with *B. subtilis*, *S. rolfsii*, and their combination, while Proteobacteria showed the opposite trend, increasing notably in the BS treatment. Other phyla, including Acidobacteriota, Bacteroidota, and Actinobacteriota, were present at lower relative abundances and exhibited varying responses to the inoculation treatments. At the bacterial genus level (Figure 6B), *Lactobacillus* was the dominant genus in the control but decreased sharply in abundance in the B, S, and BS treatments. Conversely, genera such as *Bacillus*, *Chujaibacter*, and *Rhodanobacter* showed increased relative abundance in the inoculated treatments, particularly in the BS group. For fungal communities, Ascomycota and Mortierellomycota were the dominant phyla across all treatments (Figure 6C). The relative abundance of Ascomycota was highest in the S treatment, while Mortierellomycota was most abundant in the B treatment. At the fungal genus level (Figure 6D), *Trichocladium* and *Fusarium* were the dominant genera in most treatments. The relative abundance of *Trichocladium* was highest in the S treatment, whereas *Fusarium* showed increased abundance in the BS treatment. *Mortierella* was present in all groups but was particularly abundant in the C and B treatments. Collectively, these results demonstrate that inoculation with *B. subtilis* and/or *S. rolfsii* significantly altered the taxonomic composition of both rhizosphere bacterial and fungal communities, with distinct shifts in dominant phyla and genera observed under different inoculation regimes.

**Figure 7:**
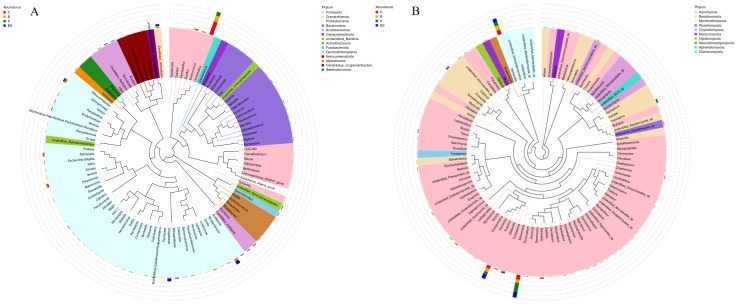
Phylogenetic trees of the top 100 genera of bacteria (**A**) and fungi (**B**) based on their relative abundance in four treatments.

Additionally, phylogenetic trees were constructed for the top 100 most abundant bacterial and fungal genera (Figure 7A,B), which clearly display the relative abundance patterns of these genera across the four soil treatments.

### 2.6. Changes in Core and Specific Microbes

**Figure 8:**
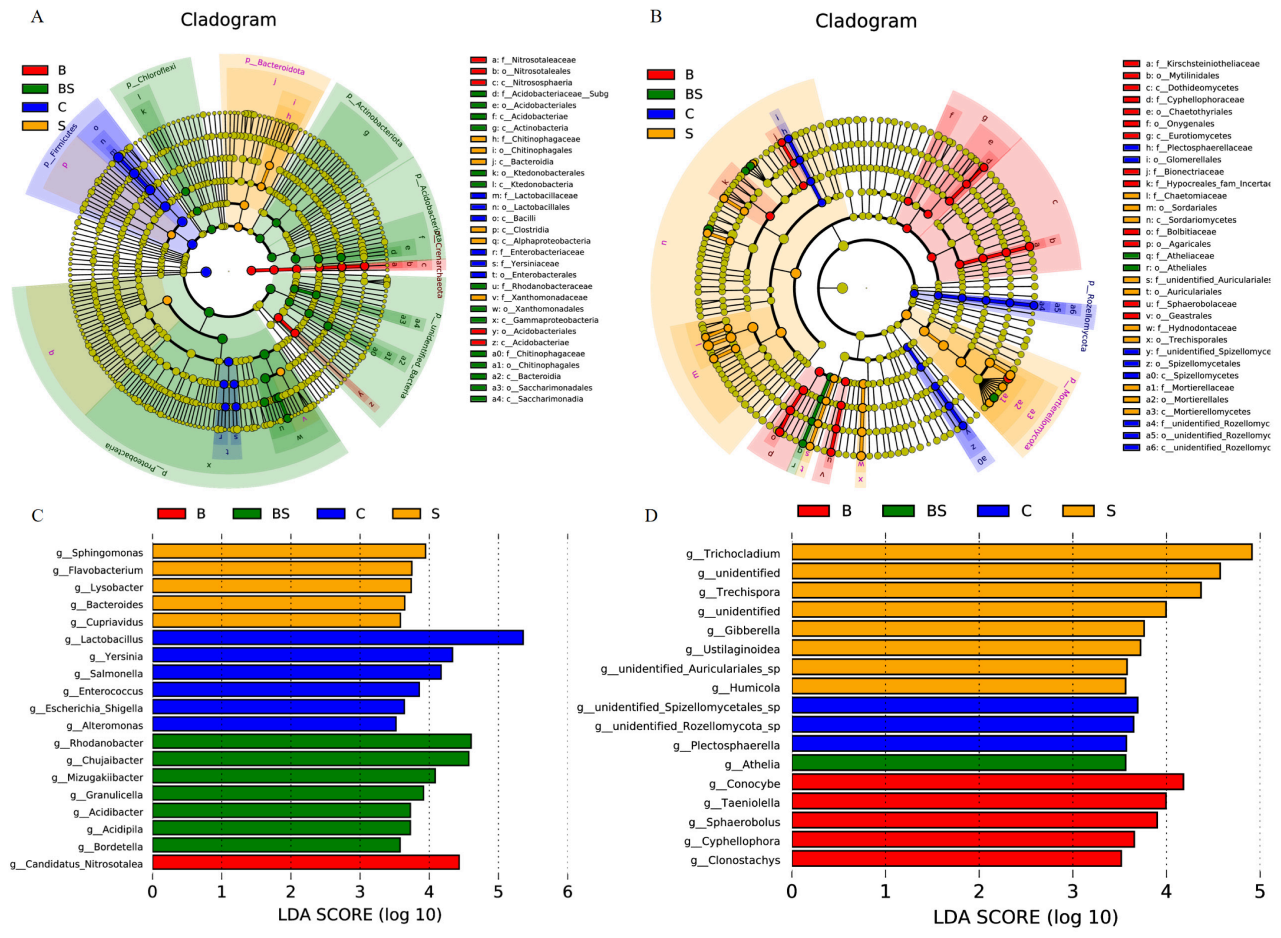
Linear discriminant analysis effect size of the rhizosphere microbial biomarkers. (**A**,**B**). Microbial core community composition of bacteria (**A**) and fungi (**B**). (**C**,**D**). LDA scores of biomarker microbiomes in bacteria (**C**) and fungi (**D**).

To identify the core and specific microbial taxa with significant differential abundance across the four treatments, Linear Discriminant Analysis Effect Size (LEfSe) was performed, with results visualized as phylogenetic branching diagrams (Figure 8A,B) and LDA score bar charts (Figure 8C,D).

The phylogenetic branching diagrams (Figure 8A for bacteria, Figure 8B for fungi) display the phylogenetic distribution of discriminative taxa at multiple taxonomic levels (phylum to genus), with colors indicating the treatment in which each taxon was most enriched. For bacteria, key biomarkers were predominantly associated with specific treatments: taxa such as *Candidatus Nitrosotalea* (order Nitrosotaleales) were significantly enriched in the B treatment; Acidobacteriales and members of the Acidobacteriaceae family were characteristic of the BS treatment; Lactobacillales (including *Lactobacillus*, Enterococcus, and related genera) were overrepresented in the C treatment; while Sphingomonadales (e.g., *Sphingomonas*), Flavobacteriales (*Flavobacterium*), and Chitinophagales were prominent in the S treatment. For fungi, distinct biomarker taxa were similarly identified: taxa within the Hypocreales, Chaetothyriales, and Mytilinidiales orders (e.g., *Clonostachys*, *Cyphhellophora*, *Taeniolella*, and *Conocybe*) were significantly enriched in the B treatment; the BS treatment showed enrichment for Atheliaceae (e.g., *Athelia*); Rozellomycota and Spizellomycota-related taxa were characteristic of the C treatment; and a broad range of Sordariales, Eurotiales, and Pleosporales taxa (e.g., *Trichocladium*, *Trechispora*, *Gibberella*, and *Humicola*) were dominant in the S treatment. The LDA score plots (Figure 8C,D) further quantified the effect size of these biomarkers, with higher LDA scores indicating stronger treatment-specific enrichment. For bacteria (Figure 8C), the most discriminative taxa were *Lactobacillus* (C treatment, LDA ≈ 5.3), *Candidatus Nitrosotalea* (B treatment, LDA ≈ 4.4), *Rhodanobacter* (BS treatment, LDA ≈ 4.6), and *Sphingomonas* (S treatment, LDA ≈ 3.9). For fungi (Figure 8D), the highest LDA scores were observed for *Trichocladium* (S treatment, LDA ≈ 4.8), *Conocybe* (B treatment, LDA ≈ 4.2), *Plectosphaerella* (C treatment, LDA ≈ 3.6), and *Athelia* (BS treatment, LDA ≈ 3.6).

Collectively, the LEfSe analysis revealed clear treatment-specific microbial signatures, with distinct bacterial and fungal taxa serving as biomarkers for each experimental condition. These findings indicate that both biocontrol inoculation and pathogen infection drive significant shifts in the rhizosphere microbiome, leading to the enrichment of unique microbial consortia associated with each treatment.

### 2.7. Correlation Analysis Between Soil Physicochemical Characteristics and Microbial Communities

**Figure 9:**
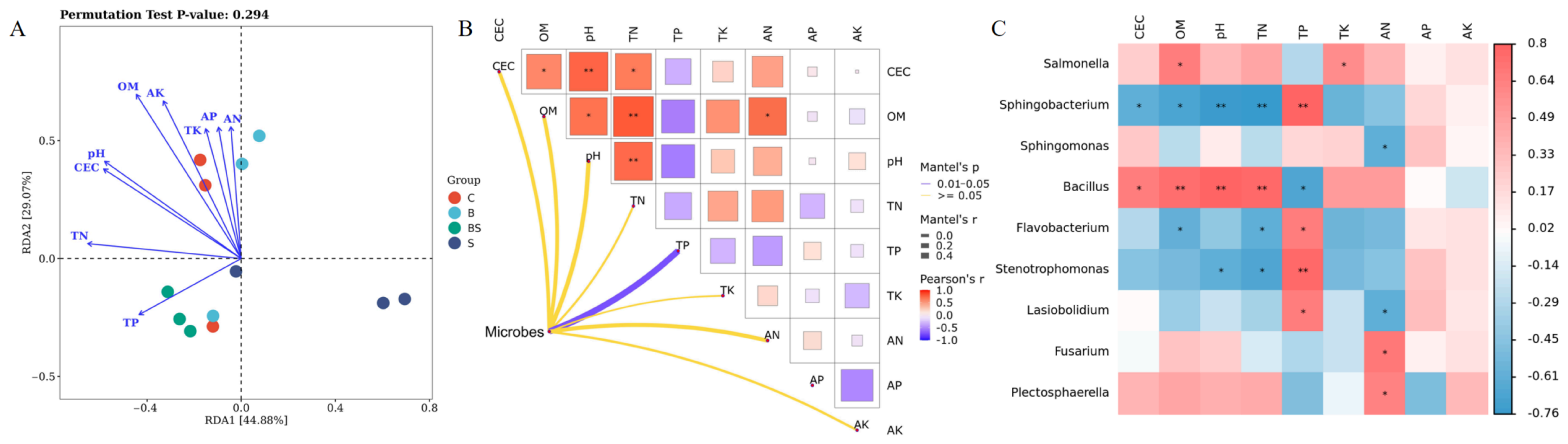
Connection between microbial communities and environmental factors. (**A**). Redundancy analysis triplots of microbial communities and soil physicochemical characteristics. (**B**). The Mantel test, which indicates the correlation between microbial community and soil properties and their pairwise comparisons through pearson correlations. (**C**). Pearson correlation coefficient diagrams for the top 20 microbial genera in relation to soil environmental factors. CEC: cation exchange capacity; OM: organic matter; TN: total nitrogen; TP: total phosphorus; TK: total potassium; AN: available nitrogen; AP: available phosphorus; AK: available potassium. * denotes significance *p* < 0.05. ** represent significance *p* < 0.01.

Redundancy analysis was applied to quantify the correlation between rhizosphere microbial community structure and soil physicochemical traits across the four inoculation treatments. The first two axes of the RDA ordination explained 44.88% (RDA1) and 29.07% (RDA2) of the total variation in microbial community composition, jointly accounting for 73.95% of total variance (Figure 9A). Samples from each treatment formed discrete clusters in the ordination space: group B samples distributed along the positive RDA1 and RDA2 axes, group S clustered far toward the positive RDA1 direction, group C located in the mid-positive RDA2 region, and BS samples gathered on the negative RDA2 side. Vectors representing soil environmental variables showed that organic matter (OM), available potassium (AK), available phosphorus (AP), available nitrogen (AN), total potassium (TK), pH, and cation exchange capacity (CEC) exhibited strong positive correlations with the RDA2 axis, while total nitrogen (TN) and total phosphorus (TP) were strongly associated with the negative RDA2 axis.

Mantel test and Pearson correlation network analyses revealed the strength and significance of associations between soil physicochemical variables and the overall microbial co-occurrence network. CEC, OM, pH, and TN displayed significant positive Pearson correlations with one another and exhibited significant Mantel correlations with the microbial network nodes (Figure 9B). TP showed a moderately strong significant Mantel correlation with the communities, while TK, AN, AP, and AK only displayed non-significant weak Mantel associations with microbial network structure. Pearson pairwise comparisons revealed robust positive inter-correlations among core nutrient and pH indicators (CEC, OM, pH, TN), forming a tightly connected environmental factor cluster that primarily shaped microbial co-occurrence patterns.

The heatmap visualized Pearson correlation coefficients between nine dominant rhizosphere microbial genera and nine soil physicochemical indices, with red shading indicating positive correlations and blue shading indicating negative correlations; asterisks mark statistically significant correlations (* *p* < 0.05, ** *p* < 0.01). The biocontrol agent *Bacillus* presented prominent significant positive correlations with CEC, OM, pH, and TN (*p* < 0.01), alongside a weak significant negative correlation with TP (*p* < 0.05). The pathogenic fungus *Fusarium* had a significant positive correlation with AN. Bacterial genera exhibited divergent responses: *Sphingobacterium* showed highly significant negative correlations with CEC, OM, pH, and TN yet a strong positive correlation with TP; *Salmonella* was positively associated with OM and TK; *Sphingomonas* had a significant negative correlation with AN; *Flavobacterium* and *Stenotrophomonas* both correlated negatively with pH and TN and positively with TP; *Lasiobolidium* had a positive link with TP and negative link with AN; the fungal genus *Plectosphaerella* displayed a significant positive correlation with AN. Collectively, key beneficial and pathogenic microbial taxa displayed contrasting response patterns to soil nutrient and pH conditions, highlighting differential environmental filtering of rhizosphere microbiota under different inoculation regimes.

### 2.8. Co-Occurrence Networks of Microbial Communities

**Figure 10:**
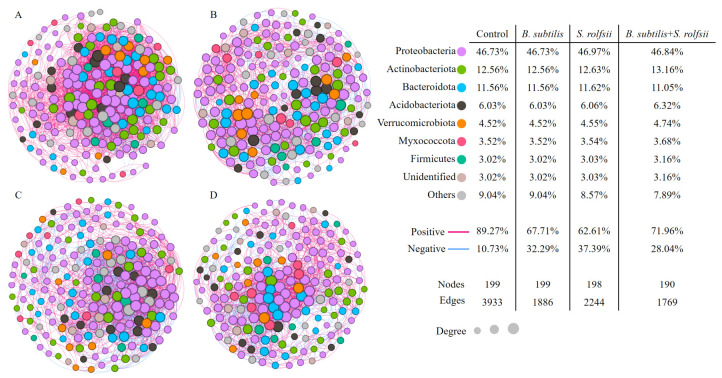
Co-occurrence networks of bacterial genera in Control (**A**), *B. subtilis* (**B**), *S. rolfsii* (**C**) and *B. subtilis* + *S. rolfsii* (**D**). The size of each node is proportional to the number of connections (degree). The color of connections between two nodes represents a positive (red) or a negative correlation (blue). The node colors show various phyla.

Co-occurrence network analysis was performed to explore the interspecies interaction patterns of rhizosphere bacterial and fungal communities across four treatments. For the bacterial co-occurrence networks (Figure 10), all networks were predominantly composed of taxa affiliated to Proteobacteria, which occupied the highest relative abundance (46.73–46.84%) across all four groups, followed by Actinobacteriota (12.56–13.16%) and Bacteroidota (11.05–11.62%). Compared with the control group (C, 199 nodes, 3933 edges), inoculation treatments remarkably reduced network scale: the numbers of total edges decreased sharply to 1886 (B), 2244 (S), and 1769 (BS), accompanied by slight declines in node quantity for the S (198 nodes) and BS (190 nodes) groups. Meanwhile, the proportion of positive correlations drastically dropped from 89.27% in the control to 67.71% (B), 62.61% (S) and 71.96% (BS), whereas the percentage of negative interactions rose accordingly, demonstrating that pathogen and biocontrol bacterial inoculation weakened the overall cooperative relationships and intensified competitive interactions within the bacterial community. Notably, co-inoculation treatment had more positive interaction in the bacterial community, suggesting a predominantly cooperative microbial relationship among genera.

**Figure 11:**
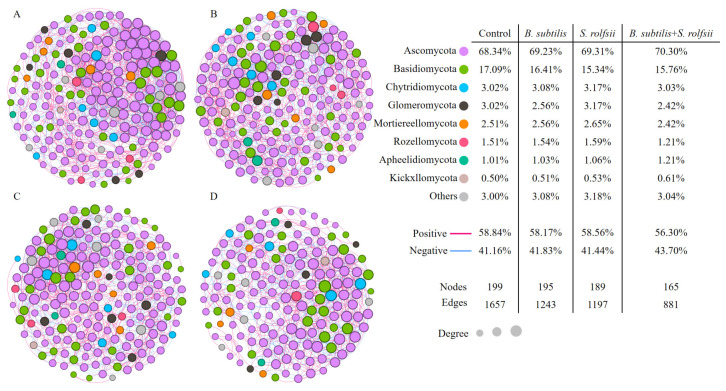
Co-occurrence networks of fungal genera in Control (**A**), *B. subtilis* (**B**), *S. rolfsii* (**C**) and *B. subtilis* + *S. rolfsii* (**D**). The size of each node is proportional to the number of connections (degree). The color of connections between two nodes represents a positive (red) or a negative correlation (blue). The node colors show various phyla.

In fungal co-occurrence networks (Figure 11), Ascomycota dominated all treatments with relative abundance ranging from 68.34% to 70.30%, followed by Basidiomycota (15.34–17.09%). Similar to the bacterial network trend, inoculation continuously simplified the topological complexity of fungal networks: total node and edge counts gradually decreased from the control (C: 199 nodes, 1657 edges) to B (195 nodes, 1243 edges), S (189 nodes, 1197 edges), and BS (165 nodes, 881 edges). The proportion of positive correlations in fungal networks fluctuated moderately and slightly declined from 58.84% (C) to 56.30% (BS), while negative correlation proportion increased from 41.16% to 43.70% under co-inoculation treatment.

Collectively, inoculation with *B. subtilis* or *S. rolfsii* simplified the topological structure of both bacterial and fungal co-occurrence networks, reduced the overall positive inter-taxa cooperation, and enhanced microbial competitive interactions, with the BS co-inoculation group showing the most obvious simplification effect on network complexity for both kingdoms.

## 3. Discussion

**Figure 12:**
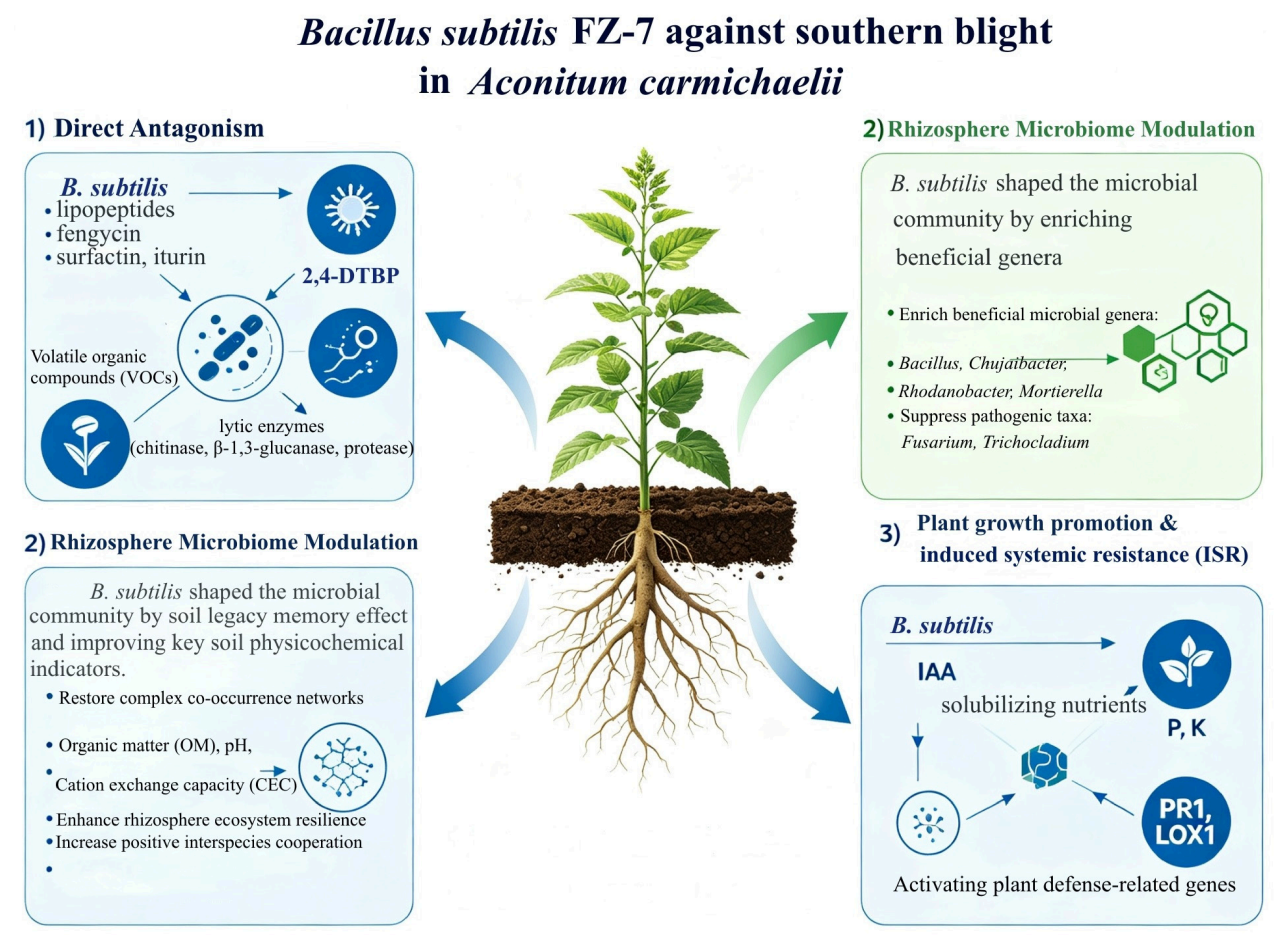
A proposed integrated biocontrol mechanism of *B. subtilis* FZ-7 against southern blight of *A. carmichaelii* caused by *S. rolfsii*.

Southern blight caused by *S. rolfsii* is one of the most destructive soil-borne diseases of *A. carmichaelii*, leading to substantial yield losses and quality deterioration [4,5]. The present study demonstrated that *B. subtilis* FZ-7 effectively suppressed southern blight and promoted plant growth under field conditions, achieving a relative control efficiency of 72.43%. Moreover, we provided novel insights into how *B. subtilis* reshapes the rhizosphere microbiome assembly, co-occurrence networks, and soil physicochemical environment to confer disease suppression. A proposed mechanistic model summarizing the multifaceted biocontrol strategies of *B. subtilis* FZ-7 is presented in Figure 12. Our findings highlight the potential of *B. subtilis* FZ-7 as a biocontrol agent that acts not only through direct antagonism but also through modulating the rhizosphere microbial community.

### 3.1. Bacillus Subtilis FZ-7 Suppresses Disease and Promotes Growth

The biocontrol efficacy of *B. subtilis* FZ-7 (72.43%) is comparable to that reported for other *Bacillus* strains against southern blight in *A. carmichaelii*. For instance, *B. velezensis* YN-2-6S achieved biocontrol efficiencies of 91.00% against southern blight in field trials [12], while *B. subtilis* JY-7-2L reduced disease severity by up to 30% with a long-acting duration of 62 days [5]. Notably, *B. velezensis* Soil-4-2 demonstrated 89.9% control efficiency in 2023 and 68.0% in 2024 under field conditions [16]. The variation in biocontrol efficacy among studies may be attributed to differences in strain characteristics, inoculation methods, soil conditions, and environmental factors. Compared with these studies, *B. subtilis* FZ-7 exhibited moderate to high control efficacy, and its ability to restore plant biomass to near-control levels underscores its practical value for sustainable *A. carmichaelii* cultivation.

Similar growth promotion by *Bacillus* strains has been reported in other medicinal plants such as *Panax ginseng* and *Ligusticum chuanxiong* [17,18]. The ability of *B. subtilis* FZ-7 to enhance plant biomass under field conditions underscores its practical value for sustainable *A. carmichaelii* cultivation.

### 3.2. Multifaceted Direct Antagonistic Mechanisms of B. subtilis FZ-7

The significant disease suppression observed in the BS treatment suggests that *B. subtilis* FZ-7 employs multiple direct antagonistic mechanisms against *S. rolfsii*. Based on the well-documented biocontrol traits of *B. subtilis* species and the observed phenotypic effects, we propose that *B. subtilis* FZ-7 likely acts through three primary direct mechanisms (Figure 12).

Firstly, production of antifungal lipopeptides. *B. subtilis* is renowned for its ability to synthesize cyclic lipopeptides belonging to the fengycin, surfactin, and iturin families, which exhibit strong antifungal activity by disrupting fungal cell membrane integrity [19]. For example, *B. subtilis* JY-7-2L, another strain isolated from *A. carmichaelii*, was confirmed to harbor bacA, fenA, fenB, fenD, srfAA, and baeA genes responsible for lipopeptide and polyketide biosynthesis [5]. Fengycin-producing *B. subtilis* SF17 achieved 74.51% control efficiency against strawberry *Fusarium* wilt, with fengycin identified as the key active metabolite [20]. Given the strong in vitro antagonistic activity of FZ-7 against *S. rolfsii* (preliminary data), it is highly probable that lipopeptide production contributes significantly to its biocontrol efficacy.

Secondly, emission of volatile organic compounds (VOCs). Recent studies have highlighted the importance of VOCs in *Bacillus*-mediated biocontrol. *B. subtilis* 0618A was found to produce 2,4-di-tert-butylphenol (2,4-DTBP) as the primary active VOC against *S. rolfsii* (EC_50_ = 0.23 μL/L), which destroys cell wall and membrane integrity, reduces mitochondrial membrane potential, and blocks ATP synthesis [21]. VOC-mediated inhibition may be particularly important for controlling sclerotia-forming pathogens like *S. rolfsii*, as volatile compounds can penetrate soil pores and reach sclerotia buried deep in the soil profile.

Thirdly, secretion of lytic enzymes. *B. subtilis* produces various hydrolytic enzymes such as chitinase, glucanase, and protease that degrade fungal cell wall components. *B. subtilis* TRO4 and CLO5 were shown to produce chitinase and β-1,3-glucanase that contribute to the inhibition of *S. rolfsii* and *Rhizoctonia solani* in chickpea [22]. These enzymes may act synergistically with lipopeptides to enhance antifungal activity.

Collectively, these direct antagonistic mechanisms likely work in concert to suppress *S. rolfsii* growth, sclerotia formation, and germination, thereby reducing disease incidence and severity.

### 3.3. Pathogen and Biocontrol Agent Differentially Modulate Rhizosphere Microbial Diversity

Alpha-diversity analysis revealed distinct responses of bacterial and fungal communities to *S. rolfsii* infection and *B. subtilis* inoculation. *S. rolfsii* infection increased bacterial alpha diversity (Shannon, Simpson) but decreased fungal diversity, while *B. subtilis* alone enhanced both bacterial and fungal richness and evenness. The increase in bacterial diversity upon pathogen infection has been observed in other pathosystems, such as ginseng rusty root and strawberry anthracnose [23,24]. This may reflect a plant-mediated recruitment of diverse bacteria to combat the invading pathogen [25,26]. However, the reduction in fungal diversity upon *S. rolfsii* infection is consistent with the idea that a single virulent pathogen can dominate the fungal niche and suppress other fungi [27]. Importantly, co-inoculation with *B. subtilis* lowered bacterial richness compared to the pathogen-alone treatment, possibly because *B. subtilis* outcompeted certain bacterial taxa or restored a more balanced community. The significantly higher fungal diversity in the *B. subtilis*-alone treatment suggests that this biocontrol agent may promote a healthier, more diverse fungal community, which is often associated with disease suppression [28,29].

Our findings align with a recent study on *B. subtilis* R31 for banana fusarium wilt control, which demonstrated that biocontrol inoculation led to a convergent enrichment of core bacterial genera such as *Burkholderia-Dyella* and *Arthrobacter-Ralstonia*, contributing to long-term disease suppression [30]. Similarly, *B. subtilis* Z-14 was found to increase microbial network linkages and enhance community stability in wheat rhizosphere infested with *Gaeumannomyces graminis* var. *tritici* [31]. These convergent observations across different pathosystems suggest that microbiome modulation is a conserved and important mechanism of *Bacillus*-mediated biocontrol.

### 3.4. Shifts in Microbial Composition: Enrichment of Beneficial Taxa and Suppression of Pathogens

PCoA and LEfSe analyses clearly separated microbial communities among the four treatments, indicating that both pathogen infection and biocontrol inoculation drive strong compositional shifts. At the bacterial phylum level, *S. rolfsii* infection reduced Firmicutes and increased Proteobacteria. This is consistent with previous studies where pathogen invasion often leads to a decline in Firmicutes (many of which are beneficial) and an increase in Proteobacteria (some of which include opportunistic pathogens) [32,33]. Conversely, *B. subtilis* inoculation enriched beneficial genera such as *Bacillus*, *Chujaibacter*, and *Rhodanobacter*. *Bacillus* is well-known for antifungal activity and plant growth promotion [8]. *Rhodanobacter* has been associated with suppression of soil-borne diseases in several crops [34]. The enrichment of *Bacillus* in the co-inoculation treatment suggests that the applied *B. subtilis* FZ-7 successfully established and proliferated in the rhizosphere, which is a prerequisite for effective biocontrol [10]. This is consistent with the findings on *B. subtilis* ED24, which successfully colonized wheat rhizosphere and persisted at high population levels throughout the growing season [35].

For fungal communities, *S. rolfsii* infection increased the relative abundance of Ascomycota and specifically enriched *Trichocladium* and *Fusarium*. *Fusarium* species are common root rot pathogens, and their increase in diseased soil is expected [4]. Notably, *B. subtilis* inoculation increased Mortierellomycota, a phylum containing many saprotrophic and plant growth-promoting fungi [36]. The genus *Conocybe* was a biomarker for the *B. subtilis*-alone treatment, and *Athelia* (the teleomorph of *S. rolfsii*) was a biomarker for the co-inoculation treatment. The presence of *Athelia* in the BS group, despite reduced disease severity, may indicate that *B. subtilis* does not completely eliminate the pathogen but rather suppresses its pathogenic activity, consistent with a biocontrol rather than eradication strategy [9].

Interestingly, our observation that *B. subtilis* enriches *Mortierella* aligns with a recent study on tomato, where combined application of humic acid, chitosan, and *B. subtilis* increased the relative abundance of *Humicola* and *Mortierella* while decreasing *Fusarium* [37]. These beneficial fungi may contribute to disease suppression through competition for resources, induction of plant resistance, or direct antagonism against pathogens.

### 3.5. Bacillus Subtilis Restores Cooperative Microbial Networks Disrupted by Pathogen Infection

Co-occurrence network analysis provided valuable insights into microbial interactions. In the uninoculated control, bacterial and fungal networks were highly complex with a high proportion of positive edges (89.27% for bacteria, 58.84% for fungi), indicating a cooperative community structure that is generally associated with soil health and disease suppression [15,38]. *S. rolfsii* infection drastically reduced the number of edges and the proportion of positive interactions, while increasing negative correlations. This suggests that pathogen invasion disrupts microbial cooperation and intensifies competition, which may weaken the soil’s natural suppressiveness [39,40]. Co-inoculation with *B. subtilis* partially restored the proportion of positive interactions (from 62.61% in S to 71.96% in BS for bacteria), indicating that the biocontrol agent helps re-establish a more cooperative microbial network. This network-level effect is an underappreciated mechanism of biocontrol and may contribute to long-term disease suppression by stabilizing the rhizosphere microbiome [13,41].

Our results are consistent with a growing body of literature demonstrating that biocontrol agents can modulate microbial network architecture. For example, *B. subtilis* combined with biochar was found to intensify bacteria-fungi interkingdom interactions and increase the abundance of beneficial modules in cut chrysanthemum rhizosphere [42]. Similarly, fengycin-producing *B. subtilis* SF17 activated microbial community structure and enhanced network complexity in strawberry rhizosphere [20]. The restoration of cooperative networks by *B. subtilis* FZ-7 may create a “buffering” effect that enhances the resilience of the rhizosphere microbiome to future pathogen invasions, a phenomenon known as “soil memory” or “legacy effect” [43].

### 3.6. Soil Physicochemical Properties Shape Microbial Community Assembly

Redundancy analysis (RDA) and Mantel tests identified soil organic matter (OM), pH, and cation exchange capacity (CEC) as the primary environmental drivers of microbial community structure. These factors are known to strongly influence microbial diversity and function [33,44]. Importantly, *Bacillus* abundance was positively correlated with OM, pH, and CEC, suggesting that *B. subtilis* FZ-7 may perform better in soils with higher organic matter and neutral pH. This has practical implications for field application: pre-conditioning soils with organic amendments or maintaining optimal pH may enhance the biocontrol efficacy of *B. subtilis* [45,46]. Additionally, *Fusarium* abundance was positively correlated with available nitrogen (AN), which aligns with reports that excessive nitrogen fertilization can exacerbate *Fusarium* diseases [47]. Therefore, integrated management combining *B. subtilis* inoculation with balanced fertilization could optimize disease control.

The interaction between soil properties and biocontrol efficacy has been documented in other systems. For instance, *B. subtilis* R31 exhibited better performance in soils with high organic matter content, which supported higher population densities and more stable colonization [30]. This suggests that the efficacy of FZ-7 may be further enhanced through soil health management practices such as organic amendment application and crop rotation.

### 3.7. Comparison with Related Studies and Implications for Biocontrol

Our results are consistent with previous reports on *Bacillus*-mediated biocontrol in *A. carmichaelii*. For instance, *B. velezensis* YN-2-6S achieved high biocontrol efficacy against southern blight (91.00%) and root rot (85.71%) through production of antifungal lipopeptides and induction of systemic resistance [12]. Similarly, *B. subtilis* FZ-7 in our study not only directly suppressed *S. rolfsii* (as evidenced by reduced disease severity) but also reshaped the rhizosphere microbiome. Unlike many laboratory-based studies, our field experiment demonstrated that the biocontrol effect persisted for at least 30 days post-inoculation, with significant recovery of plant biomass. The combination of plant growth promotion and microbiome modulation makes *B. subtilis* FZ-7 a promising candidate for integrated disease management.

However, several questions remain. Firstly, the specific antifungal metabolites produced by *B. subtilis* FZ-7 were not characterized in this study. Future work should identify the lipopeptides (e.g., surfactin, fengycin, iturin) or volatile compounds responsible for direct pathogen inhibition, using approaches such as LC-MS/MS and GC-MS. Whole-genome sequencing of *B. subtilis* FZ-7 would also provide valuable insights into its biosynthetic potential for secondary metabolites, as demonstrated for *B. subtilis* Z-14 which harbored 10 secondary metabolite gene clusters with antimicrobial activity [31]. Secondly, while we observed shifts in microbial community composition, we did not measure functional genes (e.g., involved in nitrogen cycling, antibiotic production). Metagenomic or metatranscriptomic analyses would provide deeper mechanistic insights. Thirdly, the long-term persistence of *B. subtilis* FZ-7 in the rhizosphere and its effects on successive cropping cycles need to be evaluated. Long-term field trials are essential to assess the durability of biocontrol efficacy and potential impacts on the indigenous soil microbiome.

In summary, our study demonstrates that *B. subtilis* JY-7 effectively controls southern blight in *A. carmichaelii* through a dual mode of action: direct pathogen suppression and indirect modulation of the rhizosphere microbiome. The biocontrol agent enhances beneficial bacterial and fungal taxa, restores cooperative microbial networks, and interacts favorably with soil physicochemical properties. These findings advance our understanding of microbiome-mediated biological control and provide a scientific basis for developing *Bacillus*-based products for sustainable production of *A. carmichaelii*. Future research should focus on field-scale validation, formulation development, and integration with other agronomic practices.

## 4. Materials and Methods

### 4.1. Field Experiment and Sample Collection

The field experiment was conducted across 24 experimental plots located in Dakang Town, Jiangyou City, Sichuan Province, China (104.82° E, 31.85° N), an area characterized by a longstanding practice of rotating *A. carmichaelii* and rice cultivation. Field topsoil characterization revealed a near-neutral pH of 6.95. The soil was rich in organic matter (35.46 g/kg) and exhibited substantial fertility, with total nutrient concentrations of 2.01 g/kg for nitrogen (N), 0.81 g/kg for phosphorus (P), and 13.24 g/kg for potassium (K). Correspondingly, available nutrient pools were abundant, measuring 201.7 mg/kg for alkali-hydrolyzable nitrogen, 71.5 mg/kg for Olsen-P, and 173.9 mg/kg for available potassium. Identical field management and agronomic routines were applied across all plots to eliminate extraneous environmental variables. Seedlings of *A. carmichaelii* were acquired from a local professional cooperative specializing in medicinal herb cultivation in Jiangyou and transplanted on 10 December 2024. The planting layout adopted 20 cm row spacing and 15 cm inter-plant spacing. *Bacillus subtilis* FZ-7 was isolated and preserved in the Industrial Crop Research Institute, Sichuan Academy of Agricultural Sciences. Four experimental groups were set up: blank control (C), single inoculation with *B*. *subtilis* (B), single inoculation with *Sclerotium rolfsii* (S), and combined co-inoculation of *S. rolfsii* and *B. subtilis* (BS). Each treatment contained six independent plots serving as biological replicates, with each plot measuring 2 m × 5 m and containing 300 plants. An aqueous suspension of *B*. *subtilis*, containing 1 × 10^7^ spores per milliliter, was administered through root irrigation at a volume of 50 mL per plant, ten days after the seedling emergence. Subsequently, ten days after the application of the biocontrol agent, approximately 30 sclerotia of *S*. *rolfsii* were inoculated at a depth of 5 cm beneath the soil surface adjacent to the root zone in the designated inoculation groups. The control group was administered equivalent volumes of sterile distilled water instead of the two microbial agents. Rhizosphere soil was sampled 60 days following pathogen inoculation, at which point disease incidence and disease index values for every treatment were recorded simultaneously. Six biological replicates were gathered per treatment; each replicate composite sample was blended from ten randomly picked individual plant subsamples. Aboveground shoots and underground rhizomes were separated to measure fresh biomass weight. These plant tissues were moved to the lab, oven-dried separately at 70 °C for 72 h, and then weighed to acquire dry biomass data, following the protocol outlined by Ren et al. [48]. Rhizosphere soil was sampled with strict sterile handling: loose bulk soil was gently shaken off root systems, retaining a thin ~1 mm soil layer adhering to root surfaces. This tightly bound rhizosphere soil was brushed off using a disinfected brush and immediately transferred into 1.5 mL centrifuge tubes. Collected soil samples were kept in insulated ice coolers and delivered to the laboratory within a 4-h window. Each soil replicate was split into two portions: one aliquot was preserved at −80 °C for subsequent total genomic DNA extraction, and the second subset was air-dried ahead of soil physicochemical property testing. Southern blight disease severity triggered by *S. rolfsii* was scored according to the classification standards developed by Li [49]. The five-tier grading scale is defined as: Grade 0: Rhizomes free of any necrotic lesions; Grade 1: Minor scattered lesions covering less than 25% of total rhizome surface area; Grade 2: Moderate lesion coverage spanning 25–50% of rhizome tissue; Grade 3: Extensive large lesions occupying 50–75% of the rhizome; Grade 4: Coalesced necrotic lesions covering 75–100% of the entire rhizome. Measured severity grades were then used to compute the final disease index via the calculation formula reported in a research [50]:

Disease index = (Σ(number of affected plants at each severity level × corresponding relative value))/(total number of plants under examined × maximum value of disease scale) × 100.

### 4.2. Physicochemical Properties of Rhizosphere Soil

Soil physicochemical properties were determined following established analytical protocols from published literature [51,52]. Soil pH was tested with a Sartorius pH meter (Shenzhen, China) using a soil-water mixture at a ratio of 1:2.5. Cation exchange capacity (CEC) was measured via the ammonium saturation diffusion method [53]. Soil organic matter (OM) content was determined using the potassium dichromate-sulfuric acid oxidation approach [54]. Total nitrogen (TN) was quantified with the semi-micro Kjeldahl digestion method [55]. After perchloric acid-sulfuric acid digestion, total phosphorus (TP) was detected by the molybdenum-antimony colorimetric assay [56]. Total potassium (TK) was analyzed using sodium fusion combined with flame atomic absorption spectrometry [57]. Alkaline permanganate titration was applied to measure available nitrogen (AN) [58]. For available phosphorus (AP), soil samples were first extracted with 0.5 M sodium bicarbonate solution (pH 8.5), then analyzed using the molybdenum-antimony colorimetric method. Measurements were performed at 700 nm with a UV-2600 ultraviolet-visible spectrophotometer (Mettler-Toledo, Shanghai, China) [59]. Available potassium (AK) was extracted with ammonium acetate solution and subsequently quantified [60].

### 4.3. DNA Extraction and Sequencing

Total genomic DNA was isolated from 250 mg rhizosphere soil samples using the DNeasy PowerSoil Kit (QIAGEN, Düsseldorf, Germany), strictly in line with the manufacturer’s standard protocols. The purity and integrity of extracted DNA were first checked via 1% agarose gel electrophoresis (120 V, 30 min), while the NanoDrop ND-2000 spectrophotometer A_260_ 1.8 (Thermo Scientific, Wilmington, DE, USA) was used to preliminarily detect DNA concentration. Furthermore, the Qubit 2.0 fluorometer (Life Technologies, Carlsbad, CA, USA) was adopted for accurate DNA quantification. All DNA samples were normalized to a concentration of 5 ng·μL^−1^ with a final volume of 20 μL, then freeze-dried and sent to Novogene Co., Ltd. (Tianjin, China) for 16S rRNA and ITS amplicon sequencing (Illumina NovaSeq 6000, PE250, San Diego, CA, USA). For bacterial analysis, the V3–V4 hypervariable region of the 16S rRNA gene was amplified using the primer pair 515F (5′-GTGCCAGCMGCCGCGGTAA-3′) and 806R (5′-GGACTACCAGGGTATCTAAT-3′) [61]. Fungal ITS1 region was amplified with the universal primers ITS1F (5′-CTTGGTCATTTAGAGGAAGTAA-3′) and ITS2 (5′-GCTGCGTTCTTCATCGATGC-3′) [62]. All amplified products were sequenced on the Illumina NovaSeq platform at Novogene following standard experimental workflows. Bioinformatic analysis of bacterial sequencing data was conducted using R 4.10. The Cutadapt plugin (v2.10) embedded in the DADA2 package was used to trim primer sequences from raw NovaSeq reads [63]. Raw sequences were processed and taxonomically annotated with the DADA2 pipeline [64]. Specifically, forward and reverse reads were trimmed to 240 bp and 200 bp respectively, and merged with a minimum overlap of 12 bp; reads containing over 18 predicted errors were discarded. Chimeric sequences were removed and sequences were dereplicated to obtain amplicon sequence variants (ASVs), which were then taxonomically annotated against the SILVA v138 database [65]. Fungal paired-end sequences were processed using QIIME2 for demultiplexing. After adapter and primer removal via Cutadapt 5.2 and quality filtering, the DADA2 tool was used for single-end denoising. The UNITE 8.0 database was used for the taxonomic identification of fungal ITS sequences [66]. ASVs were classified at a 97% sequence similarity threshold, and the resulting data were converted into BIOM format. Only ASVs containing no fewer than ten reads were retained for subsequent analyses.

### 4.4. Statistical Analysis

Statistical differences among four groups were examined using Duncan’s multiple range test, where a *p*-value below 0.05 was defined as statistically significant. All other statistical analyses were run in R version 4.2.1. Alpha diversity indices including Chao1, ACE, Shannon and Simpson were calculated to evaluate the richness and diversity of microbial communities, as well as the shared and unique OTUs across samples [67]. Beta diversity based on Bray-Curtis distance was applied to characterize microbial community composition and structure, and the results were visualized via non-metric multidimensional scaling (NMDS) [68]. The vegan package in R was used to perform Principal Component Analysis (PCA), Principal Coordinates Analysis (PCoA) and Redundancy Analysis. Analysis of Similarities (ANOSIM) was also carried out with the Bray-Curtis distance matrix following the protocol described by Sun et al. [69]. Pearson correlation and linear regression analyses were adopted to explore the associations between disease index and key microbial taxa. Linear discriminant analysis effect size (LEfSe) was used to screen differentially abundant OTUs [70]. Mantel tests were implemented via the vegan package to clarify correlations between soil physicochemical properties and microbial communities. Pearson correlation analysis was performed to analyze the relationships among various soil parameters, and the outcomes were presented as correlation heatmaps. A significance level of *p* < 0.05 was adopted for all statistical tests. Microbial co-occurrence networks for healthy and diseased groups were constructed using the Hmisc package in R 4.2.1, referring to previously reported methods [71,72,73]. Spearman correlation coefficients were used to filter valid correlations: only pairs with coefficients > 0.8 or <−0.7 and adjusted *p* < 0.05 (Benjamini-Hochberg correction) were retained for network construction. The topological properties of the resulting networks were analyzed according to published approaches [15].

## 5. Conclusions

This study demonstrates that *B. subtilis* FZ-7 effectively controls southern blight in *A. carmichaelii* through both direct disease suppression and indirect rhizosphere microbiome modulation. Inoculation with *B. subtilis* FZ-7 reduced disease incidence and index by 63.8% and 55.3, respectively, and largely restored pathogen-impaired plant biomass to near-control levels, confirming its strong biocontrol and growth-promoting efficacy.

Mechanistically, *S. rolfsii* infection disrupted the rhizosphere microbiome by decreasing fungal diversity, simplifying co-occurrence networks, and enriching pathogenic taxa such as *Fusarium* and *Trichocladium*. In contrast, *B. subtilis* FZ-7 inoculation reshaped the microbial community by enriching beneficial genera including *Bacillus*, *Chujaibacter*, and *Rhodanobacter*, partially restoring network complexity and cooperative interactions, and maintaining a more stable rhizosphere ecosystem. Soil organic matter, pH, and cation exchange capacity were identified as key environmental drivers shaping these microbial shifts, with *Bacillus* abundance positively correlated with these factors.

Collectively, these findings indicate that the biocontrol efficacy of *B. subtilis* FZ-7 extends beyond direct antagonism to encompass microbiome-mediated ecological regulation. This study provides a theoretical basis for developing *Bacillus*-based biocontrol strategies for sustainable management of southern blight in medicinal plant cultivation.

## Figures and Tables

**Table 1:** Disease index of Chinese cabbage clubroot under different treatments.

Treatment	Group	Disease Incidence (%)	Disease Index	Relative Control Efficiency (%)
Control	C	0.00 ± 0.00 c	0.00 ± 0.00 c	-
*S. rolfsii*	S	100.00 ± 0.00 a	76.29 ± 2.37a	-
*B. subtilis*	B	0.00 ± 0.00 c	0.00 ± 0.00 c	-
*S. rolfsii* + *B. subtilis*	BS	36.22 ± 4.25 b	21.03 ± 2.18 b	72.43 ± 2.55

Different lowercase letters indicate significant differences at *p* < 0.05 (Duncan’s test).

## Data Availability

Data will be made available on request.

## Supplementary Materials

The following supporting information can be downloaded at: https://www.mdpi.com/article/10.3390/plants15152308/s1, Figure S1: Plant rhizoma growth characteristics and the occurrence of southern blight in *Aconitum carmichaelii* from four groups. A. The control (C group); B. Single inoculation with *Bacillus subtilis* (B group); C. Single inoculation with *Sclerotium rolfsii* (S group); D. Co-inoculation of *S. rolfsii* and *B. subtilis* (BS group).
