# Supplementary material for: Effects of an Isolate of Bacillus subtilis on Southern Blight Severity of Aconitum carmichaelii Debeaux and Its Rhizosphere Microbial Community
Source: Plants (Basel). 2026 Jul 27;15(15):2308. doi: 10.3390/plants15152308 (PMC13468171; doi:10.3390/plants15152308)
Supplement: Supplementary file 1 [file plants-15-02308-s001.zip › plants-4421762-supplementary.pdf]

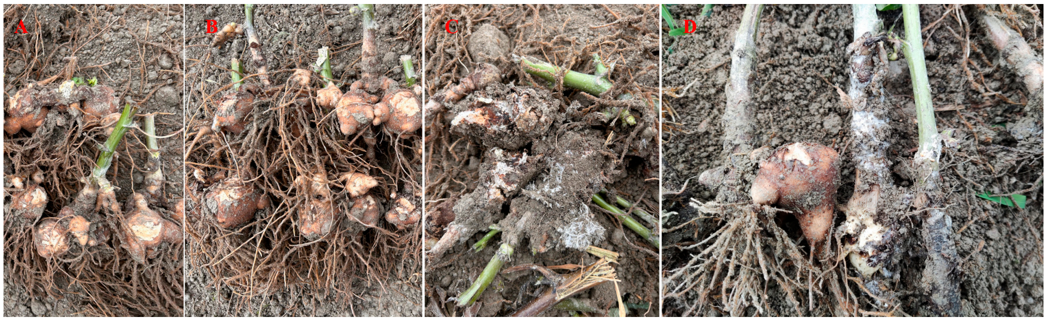

Figure S1. Plant rhizoma growth characteristics and the occurrence of southern blight in *Aconitum carmichaelii* from four groups. A. The control (C group); B. Single inoculation with *Bacillus subtilis* (B group); C. Single inoculation with *Sclerotium rolfsii* (S group); D. Co-inoculation of *S. rolfsii* and *B. subtilis* (BS group).
